# A Single Centre Experience With Routine Magnetic Resonance Cholangiopancreatography in the Management of Patients With Gall Stone Disease

**DOI:** 10.7759/cureus.18743

**Published:** 2021-10-13

**Authors:** Tanmay Pareek, Rajkumar R, Prabhakaran R, Sugumar Chidambaranathan, Naganath Babu O L

**Affiliations:** 1 Surgical Gastroenterology, Madras Medical College, Chennai, IND

**Keywords:** silent stone, common bile duct stone, laparoscopic cholecystectomy, mrcp, gall stone disease

## Abstract

Aim: To evaluate the role of preoperative magnetic resonance cholangiopancreatography (MRCP) in detection of common bile duct stone (CBDS) in cases of gall stone disease (GSD).

Methods: This is a retrospective study with a prospectively maintained database, carried out in 116 patients who underwent laparoscopic cholecystectomy (LC) for GSD, from October 2017 to September 2020. Preoperative MRCP was performed in all cases.

Results: MRCP detected CBDS in 23 out of 116 patients (19.8%) including silent CBDS in seven patients (6%). In situations of normal biochemical parameters and USG abdomen, 30.4% unnoticed CBDS out of all 23 CBDS, were discovered by MRCP. The sensitivity and specificity of aspartate aminotransferase (AST) or alanine aminotransferase (ALT) [positive predictive value (PPV): 24%; negative predictive value (NPV): 81.3%], alkaline phosphatase (ALP) (PPV: 63.2%; NPV: 88.7%), serum total bilirubin (PPV: 57.1%; NPV: 88.4%) and CBD diameter (PPV: 61.5%; NPV: 85.4%) were, respectively, 26.1% and 79.6%, 52.2% and 92.5%, 52.2% and 90.3%, and 34.8% and 94.6%. Cystic duct variations found in nine patients (7.75%). There was no bile duct injury (0%) noted in post operative patients.

Conclusion: With normal biochemical and USG parameters, MRCP is a valuable non-invasive investigation to detect the overlooked CBDS. After recognising the cystic duct variants, it may be possible to prevent bile duct injury. Before performing a laparoscopic cholecystectomy (LC) in GSD, a routine preoperative MRCP is highly recommended.

## Introduction

Gall stone disease (GSD) is one of the most common surgical problems in the world. Laparoscopic cholecystectomy (LC) has become the gold standard procedure for the treatment of gallstones [1]. However, during treatment, common bile duct (CBD) stones (CBDS) are often easily overlooked. CBDS are detected in 11%-25% of patients with gall bladder (GB) stones [2,3]. By missing CBDS following LC, there may be a chance of cystic duct blow out, bile leak, cholangitis, jaundice, and pancreatitis [3] which will cause increased morbidity, hospital stay, cost of the patient and will add the risk of litigation as well. So it is better to diagnose and treat the CBDS before LC to deliver an appropriate and cost effective treatment [4]. The main preoperative approaches for diagnosing patients with gallstones are medical history, clinical examination, liver function tests (LFTs), and abdominal ultrasonography (USG). This method, however, does not provide a reliable and precise diagnosis of CBDS [5-7]. The accuracy and sensitivity of elevated liver enzymes and abdominal USG in the diagnosis of CBDS associated with GB stone are not high [8,9]. Abdominal USG is usually used to screen these individuals for CBDS. Unfortunately, the distal part of CBD and ampulla of Vater is generally the most difficult location to observe due to intestinal gas, making identification of distal CBDS challenging [10].

CBDS may be asymptomatic, but it raises the risk of problems such as recurrent biliary colic, ascending cholangitis, and pancreatitis, thus there should be a simple way to determine the presence of CBDS prior to surgery to avoid these issues [3]. The anatomy of the biliary tract is usually well known but anatomical variations are common, which is one of the major risk factors of bile duct injury (BDI). Preoperative magnetic resonance cholangiopancreatography (MRCP) allows the surgical team to identify dangerous anatomical issues before LC with a complete evaluation of the bile duct anatomy. Preoperative detection of an accessory bile duct, an aberrant hepatic duct, or an unusual cystic duct entry may thus increase the surgical procedure's safety. Thus, identifying the exact anatomy before the surgery plays a significant role in achieving a safe surgery. The biliary and pancreatic ducts are visualised using MRCP, which is a non-invasive procedure with no associated morbidity. Although MRCP has become the gold standard diagnostic modality for CBDS [11], the routine use of pre-operative MRCP remains a point of debate. As a result, this study was carried out to evaluate its role in the management of GSD.

## Materials and methods

This is a retrospective study that included 116 patients who underwent laparoscopic cholecystectomy for GSD, from October 2017 to September 2020 at Rajiv Gandhi Government General Hospital, Chennai, India. Routine preoperative imaging was done with ultrasound abdomen and MRCP. The images were assessed for the presence of CBDS and gall bladder stones. The MRCP images were considered positive when a signal void was seen in at least two planes following the axial course of CBD. The anatomy of the biliary tree was also assessed for variation in cystic duct anatomy. Data collected included preoperative laboratory findings like bilirubin, alkaline phosphatase (ALP), alanine aminotransferase (ALT), aspartate aminotransferase (AST), amylase, lipase, white blood cell count and intraoperative presence of inflammation and complications. Patients diagnosed with CBDS were subjected to preoperative endoscopic retrograde cholangiopancreatography (ERCP) followed by LC.

## Results

In the above-mentioned period, 116 patients had undergone for cholecystectomy for GSD. Seventy-five of these patients were female (64.7%) and 41 patients were male (35.3%). Their ages ranged from 15 to 68 years, with a mean age of 40.55 years. All of these individuals had an abdominal USG and MRCP. There were 23 individuals who had both gallstones and CBDS, accounting for 19.8% of all cases. Patients had a biochemical assessment at admission, with the following preoperative laboratory findings: there were 25 patients with raised AST or ALT, [AST (45-242U/L) and ALT (44-263 U/L)], 19 patients with elevated ALP (161-700 U/L) and 21 patients with elevated serum total bilirubin (1.8-14mg/dl).

USG abdomen showed dilated CBD (>6mm) in 13 patients, of which eight patients (61.5%) had CBDS while 103 patients with normal CBD diameter had CBDS in 15 patients (14.6%). Table 1 shows the association between LFT parameters, the USG abdomen, and CBDS.

**Table 1:** Association between laboratory parameters, common bile duct diameter on USG with CBDS. AST: aspartate aminotransferase, ALT: alanine aminotransferase, ALP: alkaline phosphatase, USG: ultrasonogram, CBDS: common bile duct stone

Laboratory parameters and USG findings in patients (n)	Patients with CBDS % (n)	Patients without CBDS % (n)
Normal AST or ALT (91)	18.9% (17)	81.3% (74)
Increased AST or ALT (25)	24% (6)	76% (19)
Normal ALP (97)	11.3% (11)	88.7% (86)
Increased ALP (19)	63.2% (12)	36.8% (7)
Normal bilirubin (95)	11.6% (11)	88.4% (84)
Increased bilirubin (21)	57.1% (12)	42.9% (9)
CBD diameter-Normal (103)	14.6% (15)	85.4% (88)
>6mm (13)	61.5% (8)	38.5% (5)

The sensitivity and specificity of AST/ALT [positive predictive value (PPV): 24%; negative predictive value (NPV): 81.3%], ALP (PPV: 63.2%; NPV: 88.7%), serum total bilirubin (PPV: 57.1%; NPV: 88.4%) and CBD diameter (PPV: 61.5%; NPV: 85.4%) were, respectively, 26.1% and 79.6%, 52.2% and 92.5%, 52.2% and 90.3%, and 34.8% and 94.6%. As a result, the likelihood of having CBDS was only 63.2%, 57.1%, and 61.5% in cases of high ALP, bilirubin, and CBD diameter (>6mm), respectively, as shown in Table 2.

**Table 2:** Sensitivity, specificity, PPV & NPV of laboratory parameters and dilated CBD on USG USG: ultrasonogram, AST: aspartate aminotransferase, ALT: alanine aminotransferase, ALP: alkaline phosphatase, PPV: positive predictive value, NPV: negative predictive value, CBD: common bile duct

Laboratory and USG findings	Sensitivity	Specificity	PPV	NPV
AST/ALT	26.1%	79.6%	24%	81.3%
ALP	52.2%	92.5%	63.2%	88.7%
Total bilirubin	52.2%	90.3%	57.1%	88.4%
CBD diameter (>6mm)	34.8%	94.6%	61.5%	85.4%

In all of the patients, MRCP was conducted, and CBDS was found in 23 of them. Sixteen patients (69.6%) were suspected of having CBDS based on laboratory parameters and USG abdominal findings, while the remaining seven patients (30.4%) had CBDS even with normal laboratory and USG results and were referred to as "silent stones," accounting for 6% of all cases. These patients had no history of previous jaundice, cholangitis, or pancreatitis. If MRCP had not been performed, these 30.4% CBDS would have been overlooked. These patients were subjected to preoperative ERCP endotherapy followed by LC.

Other MRCP findings that influenced surgical approach included double cystic duct in one patient, spiral course with medial insertion of cystic duct in two patients, parallel course in three patients, high insertion near the hilum in one patient, and low insertion of cystic duct near distal CBD in two patients as depicted in Table 3. In these cases, the cystic ducts were meticulously followed, and clipping was done securely after defining the structures. Lap converted to open cholecystectomy was done in four patients. There was no bile duct injury (0%) observed in our study.

**Table 3:** Cystic duct variations on MRCP MRCP: magnetic resonance cholangiopancreatography, CBD: common bile duct

MRCP Findings	Frequency
CBD stone	23
Cystic duct variations	
Double cystic duct	1
Spiral course with medial insertion	2
Parallel course	3
High insertion (near confluence)	1
Low insertion	2

## Discussion

CBDS are found in 11% to 25% of patients with gall stone disease [1] with an incidence of unsuspected CBDS of up to 5% [11]. CBDS were found in 19.8% of patients in our study, which is consistent with prior studies. Silent CBD stones [12] were found in 6% of patients.

According to many studies, if preoperative laboratory findings and USG evaluation of CBD diameter are positive then it increases the possibility of CBD stones up to 99% [13]. Liver function tests are neither highly sensitive nor specific in screening for CBDS [2]. Previous research [14] found that bilirubin and alkaline phosphatase were the best predictors of CBD stones. However, in prior research, the sensitivity and positive predictive value of all laboratory data were modest; about 40% and 30-60%, respectively [13,15]. Our findings indicated sensitivity, PPV of serum bilirubin, and alkaline phosphatase of 52.2%, 57.1%, and 52.2%, 63.2%, respectively.

USG is widely used as first-line investigation to diagnose many biliary diseases and a CBD diameter greater than 6mm on USG is associated with a higher prevalence of CBDS [16]. However, USG has a limited sensitivity (33-55%) for detecting CBD stones [15]. The sensitivity of the USG was 34.8% in our study, which is consistent with earlier studies. The fact that USG is an operator-dependent approach, explains the presence of sensitivity variability.

Computed tomography is associated with ionising radiation and is not sensitive for identifying the non-calcified stones. ERCP is not recommended for diagnostic purposes because of its associated complications including pancreatitis, cholangitis, perforation, and bleeding and it has a morbidity rate of 3-10% and a mortality rate of 0.1-3% [13]. ERCP can be performed as a therapeutic measure in cases of CBDS for stone retrieval. Intravenous cholangiography [17] has the same sensitivity and specificity as ERCP, but it is a more invasive procedure that uses ionising radiation.

MRCP is a non-invasive procedure that does not use ionising radiation. It is an excellent tool for identifying biliary anatomy and pathology with sensitivity and specificity that matches ERCP [12,15]. In our investigation, MRCP was used in all cases, and CBDS was found in 23 individuals (19.8%), seven of whom had silent stones (6%). Similarly, two separate groups of investigators discovered silent stones in 4% and 6% of their populations, respectively, and advocated MRCP as a screening procedure before LC [12,18].

The identification of silent stone in seven patients (30.4%) out of 23 CBDS cases influenced their management and they required a preoperative ERCP procedure to avoid postoperative sequelae such as bile leak, recurrent biliary colic, cholangitis, and pancreatitis due to retained stone [3]. Due to the lack of abnormal USG findings and laboratory signs, 30.4% of the stones would have gone unnoticed. It may have had serious implications for patients as well as increased total healthcare expenses [19].

Cystic duct variations were diagnosed preoperatively in nine patients (7.7%), which is in line with previous studies [18]. These findings allowed the surgeon to be more cautious during surgery through careful dissection to identify variant anatomy and proper CBD insertion and clipping. In the series published by Ausch et al. [18], more variants of the cystic duct were detected (9.5%) and they opined that pre-operative detection of cystic duct variations is helpful in preventing bile duct injury. In our study, there was no bile duct injury (0%). In a broad national US survey from 1993, bile duct injury occurred in 0.6% of patients [20]. Nebiker et al. [12] observed a rate of bile duct injury of 0.1% between 1990 and 2002.

Romagnuolo et al. [21] presented an authoritative meta-analysis of 67 published controlled studies in 2003, concluding that MRCP has a 95% overall sensitivity and a 97% specificity for detecting CBD stones. This study does not examine the cost effectiveness for patients; nonetheless, the importance of preoperative MRCP in screening patients with gall stone disease has been evident in this study.

## Conclusions

Preoperative MRCP is a reliable non-invasive imaging study for the detection of CBDS. In our study, with normal biochemical parameters like normal AST or ALT, ALP, total bilirubin, CBD diameter, CBDS were present in 18.9%, 11.3%, 11.6%, 14.6% of cases respectively. MRCP improves the chance of detecting overlooked CBDS by 30.4%, affects the line of management, and reduces probable post-operative complications. By recognising cystic duct variations, it assists to lower the incidence of CBD injury. Thus, in gallstone disease, MRCP is suggested routinely prior to laparoscopic cholecystectomy.
